# Effect of Exercise and Oral Niacinamide Mononucleotide on Improving Mitochondrial Autophagy in Alzheimer’s Disease

**DOI:** 10.3390/nu15132851

**Published:** 2023-06-23

**Authors:** Weijia Wu, Shunling Yuan, Yingzhe Tang, Xiangyuan Meng, Mei Peng, Zelin Hu, Wenfeng Liu

**Affiliations:** 1 Hunan Provincial Key Laboratory of Physical Fitness and Sports Rehabilitation, Hunan Normal University, Changsha 410012, China; 13080535713@163.com (W.W.); yasashiyoyo@yeah.net (S.Y.); mengxiangyuan188@163.com (X.M.); hzlttxsh@163.com.com (Z.H.); 2 Key Laboratory of Protein Chemistry and Developmental Biology of Ministry of Education, Hunan Normal University, Changsha 410081, China

**Keywords:** NAD^+^, NMN, exercise, AD, mitochondrial autophagy

## Abstract

Oral niacinamide mononucleotide (NMN) and aerobic exercise have been shown to enhance niacinamide adenine dinucleotide (NAD^+^) in the body. NAD^+^ plays a critical role in the body and can directly and indirectly affect many key cellular functions, including metabolic pathways, DNA repair, chromatin remodeling, cell aging, and immune cell function. It is noteworthy that the level of NAD^+^ decreases gradually with increasing age. Decreased levels of NAD^+^ have been causally associated with a number of diseases associated with aging, including cognitive decline, cancer, metabolic diseases, sarcopenia, and frailty. Many diseases related to aging can be slowed down or even reversed by restoring NAD^+^ levels. For example, oral NMN or exercise to increase NAD^+^ levels in APP/PS1 mice have been proven to improve mitochondrial autophagy, but currently, there is no regimen combining oral NMN with exercise. This review summarizes recent studies on the effect of oral NMN on the enhancement of NAD^+^ in vivo and the improvements in mitochondrial autophagy abnormalities in AD through aerobic exercise, focusing on (1) how oral NMN improves the internal NAD^+^ level; (2) how exercise regulates the content of NAD^+^ in the body; (3) the relationship between exercise activation of NAD^+^ and AMPK; (4) how SIRT1 is regulated by NAD^+^ and AMPK and activates PGC-1α to mediate mitochondrial autophagy through changes in mitochondrial dynamics. By summarizing the results of the above four aspects, and combined with the synthesis of NAD^+^ in vivo, we can infer how exercise elevates the level of NAD^+^ in vivo to mediate mitochondrial autophagy, so as to propose a new hypothesis that exercise interferes with Alzheimer’s disease (AD).

## 1. Introduction

Alzheimer’s disease (AD) is widespread worldwide, with 6.7 million Americans aged 65 and older diagnosed with Alzheimer’s dementia in the United States alone in 2023, a number that could grow to 13.8 million by 2060 [1]. The cause of AD is still unclear, and the more popular hypotheses include the amyloid (Aβ) hypothesis, the neuronal entanglement hypothesis, and the mitochondrial cascade hypothesis, which have the common feature of suggesting that Aβ has a damaging effect on neurons [2,3]. Aβ is deposited over time, and its toxicity causes damage to the mitochondria in the neuronal cells in the brain in many ways, such as structural damage, mitochondrial malfunction, and abnormal mitochondrial autophagy [4]. The inability to remove damaged mitochondria from the brain leads to an excessive release of ROS, which contributes to inflammation, so normalizing mitochondrial autophagy appears to be a therapeutic approach to save AD neurons [5,6].

Nicotinamide mononucleotide (NMN), known as β-nicotinamide mononucleotide, is a biologically active nucleotide that is naturally formed via the reaction of a phosphate group with a nucleoside containing ribose and nicotinamide [7]. In recent years, in an animal model, an experiment confirmed that oral NMN can mitigate the negative effects of aging by increasing the amount of NAD^+^ in the body via the promotion of NAD^+^ synthesis [8]. The electron chain in mitochondria undergoing oxidative phosphorylation plays a role in transferring H^+^, and a large body of evidence indicates that NAD^+^ decreases significantly in the aging organism, resulting in the limitation of its response when the level of NAD^+^ cannot meet the demand for redox in oxidative phosphorylation, which explains why the energy metabolism is much lower in the elderly than in the young. As a result, the cells cannot obtain enough energy supply, meaning that the cells cannot carry out normal physiological functions [9,10,11]. NAD^+^ plays an important role in regulating many aspects of mitochondria, such as regulating mitochondrial homeostasis (mitophagy and autophagy), increasing mitochondrial respiration rate, and playing a key role in the pathogenesis of neurodegenerative diseases [11,12,13]. Evandro found that NAD^+^ has the ability to induce mitochondrial autophagy in the AD brain and that mitochondrial autophagy inhibits the sustained increase in Aβ and tau proteins and acts as a scavenger, reversing cognitive dysfunction in APP/PS1 mice [14,15]. Thus, oral administration of NMN to elevate NAD^+^ in vivo seems to provide a new avenue for the treatment of AD.

Many studies have shown that low-to-moderate-intensity aerobic exercise elevates NAD^+^ in vivo, and exercise has a significant effect on improving AD cognitive dysfunction [15,16]. It has been suggested that exercise also activates AMPK when it elevates NAD^+^ in vivo, which, in turn, regulates mitochondrial autophagy [17]. However, the exact mechanism by which exercise elevates NAD^+^ levels in various parts of the body is not known, and the exact pathway by which AMPK mediates mitochondrial autophagy is also not known. This review analyzes whether there is an intrinsic link between oral NMN elevation of NAD^+^ and exercise elevation of NAD^+^ in vivo and investigates how NAD^+^ affects mitochondrial autophagy to improve AD cognitive dysfunction.

## 2. Oral NMN and Exercise Enhance NAD^+^ In Vivo

### 2.1. Pathway of NAD^+^ Synthesis by NMN

The accumulation of NAD^+^ in different cellular regions is referred to as “NAD^+^ pools”, so the pathway of NMN synthesis or the enzymes that promote NAD^+^ synthesis also differ in different regions [18]. Recently, it was shown that SLC12A8, a protein that transports NMN across the cell membrane, is expressed in the small intestine, liver, and hippocampus, with the highest expression in the small intestine [19]. NMN enters the cell stroma via SLC12A8 and is synthesized via the NAD^+^ salvage pathway in the presence of NMNAT1, NMNAT2, and NMNAT3. Part of the NAD^+^ in the cell matrix remains in there, while part enters the nucleus, and another part enters the mitochondria. The NAD^+^ entering the nucleus is degraded to NAM by NAD^+^-dependent deacetylases, such as SIRT1, SIRT6, and SIRT7 and PARPs poly (ADP-ribosyl) polymerase, and then NMN is synthesized by iNAMPT. NMNAT1 in the nucleus synthesizes NMN to NAD^+^, thus completing the NAD^+^ salvage pathway in the nucleus.

SLC25A51 on the outer mitochondrial membrane has been identified in several experiments as a transporter protein for NAD^+^ [20]. NAD^+^ in the cell matrix is transported into the mitochondria by SLC25A51 and is degraded to NAM by SIRT3, SIRT4, and SIRT5, but the NAD^+^ salvage pathway in the mitochondria is unknown, and it has not been determined which of the NAMPTs is responsible for completing the conversion of NAM to NMN. Although NMN can be detected in mitochondria, whether it is converted from NAM or enters the mitochondria from NMN in the cell matrix is not known for the time being. A role for a specific NMNAT isoform (NMNAT3) has been proposed, but it is not certain that NNMN can be converted into NAD^+^ in mitochondria [21].

In summary, NMN synthesis of NAD^+^ is dominated by the NAD^+^ salvage pathway and is primarily synthesized by the NAD^+^ salvage pathway located in the extracellular fluid and the cell matrix.

### 2.2. Oral NMN Can Improve the Level of NAD^+^ in All Tissues

Oral administration of NMN can effectively enhance NAD^+^ in humans and animal models, as demonstrated in several experiments [22,23,24]. NAD^+^, as a regulatory factor closely related to energy metabolism, is, therefore, present in various tissues, but its expression varies greatly in different tissues. In rodent studies, oral or injected NMN effectively enhances NAD^+^ biosynthesis in a variety of tissues, including the pancreas, liver, adipose tissue, heart, skeletal muscle, and kidneys, and the NAD^+^ levels in the hippocampal and hypothalamic brain regions also rapidly increase [25,26,27,28,29,30,31,32,33,34,35]. The above results suggest that NMN can pass through the BBB and act as a substrate for NAD^+^ biosynthesis in brain regions, providing one piece of evidence that NAD^+^ can ameliorate neurodegenerative diseases.

The highest expression of SLC12A8, the transporter protein of NMN, has been found in the small intestine, so the most significant increase in NAD^+^ has been detected in the small intestine after NMN administration [19,36]. Alessia et al. used double-labeled isotope NMN (O18-D-NMN) fed to WT mice and clearly detected 018-D-NMN in the jejunum and ileum after 10 min [19]. NMN in the small intestine was absorbed into the blood through SLC12A8 on the small intestinal villi. Additionally, in the experiments of Alessia et al., who fed NMN at 500 mg/kg body weight to WT-type mice, the plasma NMN levels significantly increased 5 min after feeding [19]. There appear to be differences in the efficiency of the intestinal absorption of different doses of NMN feeding. In a study by Kathryn et al., feeding ET mice at 300 mg/kg body weight resulted in a rapid increase in the plasma NAD^+^ levels at 2.5 min, with a sustained increase during the 5–10 min period and a return to original levels at 15 min [24]. In experiments using humans as study subjects, similar results to those obtained in rodents have been observed, with oral administration of NMN resulting in similarly elevated plasma NMN and NAD^+^ concentrations. Back in 1995, Ann et al. found a rapid increase in the plasma levels of nicotinamide in a trial in which young men were given low (2.5 mg/kg body weight) and high (25 mg/kg body weight) doses of nicotinamide, with the increase being more rapid with higher doses [37]. In a Japanese clinical trial of oral NMN in 2020, significant changes in plasma NMN metabolites were found in adult men taking 100, 250, or 500 mg NMN capsules orally, with the most pronounced changes occurring at a dose of 500 mg/kg [22].

The liver contains most of the enzymes required in the NAD^+^ biosynthetic pathway, and Liu et al. showed that the liver accounts for more than 95% of circulating nicotinamide in mice using isotope tracer techniques and quantitative flux analysis, suggesting that the main site of NMN conversion to NAD^+^ via the biosynthetic pathway is in the liver [36]. In Kathryn et al.’s study, mice were fed double-labeled isotopic NMN (C13-D-NMN) at a dose of 300 mg/kg body weight, and using mass spectrometry to track these markers in the liver NAD^+^, it was found that although the increase in NMN in the liver was not as pronounced and rapid as in the blood, double-labeled NAD^+^ was clearly detected at 13 min (C10-D-NAD^+^), with a further increase in C30-D-NAD^+^ levels at 13 min [24].

Oral or injected NMN similarly elevates NAD^+^ water in muscles [24,38]. Additionally, in the experiments of Kathryn et al., C30-D-NAD^+^ was detected in mouse flounder muscle 13 min after feeding [24]. In Golam et al.’s experiment, they injected a dose of 500 mg/kg body weight from the peritoneal cavity of mice and found elevated levels of NAD^+^ in mouse muscle [38]. In a human experiment with elderly subjects, 250 mg of NMN was administered to elderly men daily for 6 or 12 weeks, and although the investigators only measured blood levels of NMN versus NAD^+^ and found a significant increase, muscle strength was found to be increased in the elderly taking NMN [38].

Elevated NAD^+^ in the brain can help improve cognitive dysfunction [39]. In a 2021 study of NMN to improve CICI-induced cognitive dysfunction, it was found that feeding C57 mice at a dose of 250 mg/kg body weight increased their NAD^+^ levels in the hippocampus [40]. Ruben et al. similarly demonstrated, in their experiments, that injecting NMN into the body is effective in boosting the NAD^+^ levels in the brain, when they administered C57BL/6N mice via injection of a dose of 250 mg/kg body weight and found an increase in the NAD^+^ levels in the brains of the mice upon taking the material and 24 h later [41]. A higher dose of NMN was tested in a trial by Chidambaram et al. They administered strong oral feeding to C57/B6J mice at a dose of 400 mg/kg body weight and examined the brain tissue from the mice 45 min after feeding, finding a significant increase in the NAD^+^ levels in their brains [8].

### 2.3. Exercise Regulates the Level of NAD^+^ In Vivo

The way in which exercise regulates NAD^+^ varies considerably between tissues, and the different intensities of exercise have different effects on NAD^+^, probably due to the existence of a more complex synthetic pathway for NAD^+^. Studies have shown that the kynurenine pathway in the liver accounts for 90% of the whole body, due to the fact that the enzymes required for the kynurenine pathway are not expressed in most extrahepatic cells [36]. Therefore, exercise appears to be more dependent on the second pathway for NAD^+^ synthesis, i.e., through redox reactions in the energy metabolism.

Several experiments have reported changes in the NAD^+^ content and NAD^+^/NADH ratio in muscle in vivo in animals (mice, rats, and insects) and humans by exercise, but the results obtained in animal and human experiments differ significantly due to the intensity of exercise [42]. In experiments with humans, it has been concluded that the NAD^+^ content has different degrees of expression with the intensity of exercise, with a decrease in the NAD^+^/NADH ratio (NAD^+^ decreases and NADH increases) at 60% and 100% of the maximum oxygen uptake (i.e., moderate- to high-intensity exercise). However, in another experiment, it was concluded that the NAD^+^/NADH ratio increases at 50% of the maximal oxygen uptake (NAD^+^ rises and NADH falls), suggesting that low-to-moderate-intensity exercise (i.e., aerobic exercise) increases NAD^+^ levels [43,44].

The reason for such a large difference in NAD^+^ expression at different exercise intensities could be that the high glycolysis rates under high-intensity exercise lead to a decrease in NAD^+^/NADH, as has been demonstrated in cardiac myocytes [45]. It is now hypothesized, with respect to high glycolysis causing a decrease in NAD^+^/NADH, that since oxidative phosphorylation proceeds depending on the availability of NADH, i.e., the balance between the reduction in NAD^+^ to NADH and the oxidation of NADH to NAD^+^, high glycolysis rates lead to NADH saturation, while the malate–aspartate and α-phosphoglycerol shuttle systems, which oxidize NADH to NAD^+^, have limited capacity, resulting in lower NAD^+^/NADH [46]. Therefore, elevating NAD^+^ in the cytoplasm and mitochondria in vivo through low-to-moderate-intensity aerobic exercise is a feasible option.

## 3. NAD^+^ Ameliorates Abnormal Mitochondrial Autophagy

### 3.1. AD Leads to Abnormal Mitochondrial Autophagy in the Brain

**Figure 1:**
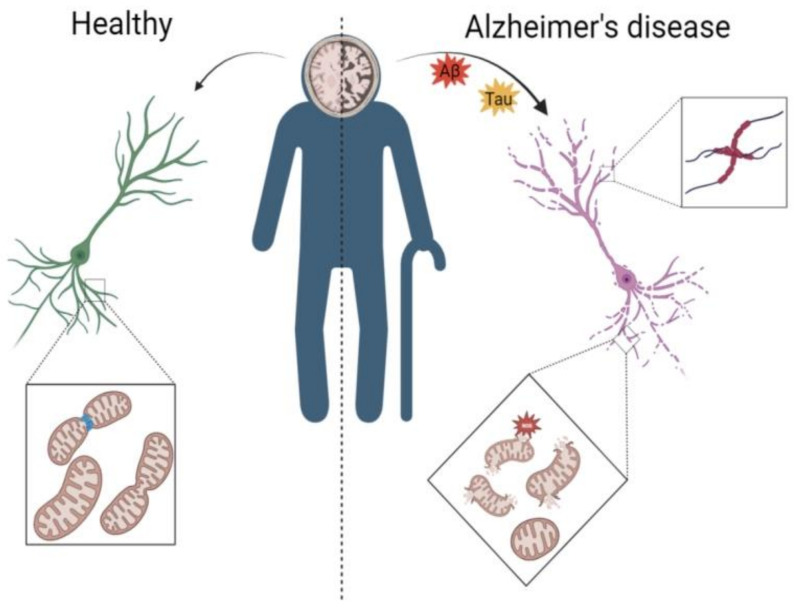
The mitochondria of the neurons in the brain of Alzheimer’s disease patients are damaged. The left side of Figure 1 shows the mitochondria of neurons in a healthy brain, with the mitochondrial kinetics and mitochondrial autophagy being in normal state; the right side of Figure 1 shows neurons in an AD brain; due to the toxicity of Aβ and Tau, the neurons become entangled, the mitochondrial kinetics and mitochondrial autophagy in neurons are abnormal, and the mitochondria break down and release ROS to further harm the neurons.

The accumulation of Aβ is one of the pathological features of AD, and it has been experimentally demonstrated that severe damage to mitochondria occurs in the AD brain. Damage to the mitochondria leads to a lack of energy supply in the brain, which prevents the clearance of Aβ [47,48]. The toxic effect of Aβ on mitochondria further impairs many mitochondrial functions, such as structural and autophagic abnormalities in the brain [49,50]. When abnormal mitochondrial autophagy is coupled with slowed energy metabolism in the AD brain, it leads to reduced AMPK activity [51,52]. Reduced AMPK expression tends to inhibit SIRT1 and PGC-1α, which regulate mitochondria-related functions, and the AMPK/SIRT1/PGC-1α pathway fails to regulate mitochondrial dynamics, leaving the mitochondrial mass uncontrolled and exacerbating abnormal mitochondrial autophagy [4]. Moreover, it has been experimentally demonstrated that when the energy metabolism is imbalanced in the brain, it causes axonal damage [53]. As shown in Figure 1, the AD brain undergoes marked atrophy and neuronal damage due to aberrant autophagy (Figure 1).

At the same time, the PINK–PARKIN pathway is inhibited in the AD brain [4]. In normal neuronal cells, PINK1 can recruit PARKIN to damaged mitochondria and can lead to the recruitment of p62 (SQSTM1) and ubiquitinated mitochondria or other autophagy-related proteins, thereby inducing mitochondrial autophagy. Mitochondria are damaged by Aβ, coupled with inhibition of the PINK–PARKIN pathway, such as proteins associated with autophagy. The activating molecules of becn1 regulatory autophagy protein 1 (AMBRA1), Bcl2L13, FUN14 domain-containing protein 1 (FUNDC1), and NFKB-1 mitochondrial ubiquitin ligase activator (MUL1)2 in neuronal cells in AD are reduced, and the levels of lipid-modified microtubule-associated protein light chain 3 (LC3B-II) and beclin-1 are lower, with both autophagosomes and autosome numbers being reduced in AD neurons [52,54]. These results suggest normal autophagic flux but reduced overall induction of the autophagic pathway. Thus, mitochondrial autophagy damage has been detected in both hippocampal samples and neurons of AD [14]. This results in abnormal mitochondrial autophagy, with severely damaged mitochondria not being cleared by autophagy, resulting in the inability of new mitochondria to be synthesized (when old mitochondria are phagocytosed by lysosomes to provide the protein material needed for new mitochondrial synthesis) [55,56].

In AD, in addition to the toxicity of Aβ and Tau, which can act directly on mitochondria, they can also cause an inflammatory response, disrupting the balance of ROS production and elimination in the brain. ROS continue to accumulate, leading to the development of an inflammatory response, along with Foxo3 acetylation, which leads to a decrease in the energy of mitochondria to resist ROS, resulting in a decrease in mitochondrial activity and an abnormal mitochondrial dynamics, i.e., a serious imbalance between mitochondrial fusion and division, leading to a decrease in the mitochondrial mass in the brain (Figure 1) [57,58,59].

### 3.2. Mechanisms by Which NAD^+^ Ameliorates Abnormal Mitochondrial Autophagy in AD

NAD^+^ is a coenzyme used in redox reactions and is a key regulator of the energy metabolism [60]. Increased intracellular and mitochondrial NAD^+^ levels maintain mitochondrial fitness and improve mitochondrial biogenesis, mitochondrial unfolded protein responses, and mitochondrial autophagy [61]. In recent years, it has been found that during aging and age-related diseases, such as AD and T2DM, the NAD^+^ levels are altered in several organs of mice and humans [24,56,62,63,64]. As there is both severe DNA damage and chronic inflammation in the AD brain, this leads to an increase in PARPs, CD38, SARM1, and a few other NAD^+^-depleting substances, further exacerbating the depletion of NAD^+^ in the brain. As Covarrublias et al. concluded in their article, when PARPs, CD38, SARM1, and some other NAD^+^-depleting substances were inhibited, NAD^+^ levels in AD were significantly increased, and AD brain dysfunction and cognitive impairment were improved [11]. Recent studies have shown that the administration of the NAD^+^ precursor NMN is rapidly absorbed and converted to NAD^+^ by nicotinamide/nicotinic acid mononucleotide adenyl transferase (NMNAT), which rapidly and effectively elevates the NAD^+^ levels in the body [65,66]. NMN supplementation may inhibit chronic diseases associated with aging; for example, in AD, NMN supplementation may improve the mitochondrial and neuronal function in the brain [35,65,67].

SIRT1 is an NAD^+^-dependent deacetylase located primarily in the nucleus, and elevated NAD^+^ levels in organisms activate SIRT1 [68]. SIRT1 has been shown to improve the mitochondrial oxidative metabolism and positively regulate autophagy and mitochondrial function in response to oxidative stress [69,70,71]. The overexpression of SIRT1 stimulates autophagosome formation and increases basal autophagy levels, while SIRT1 deficiency prevents autophagy during nutrient deprivation [70,72,73]. In the latest study by Rasti et al., SIRT1 was the main factor of DNA damage response and DNA repair, autophagy could also be understood as a response to DNA damage, and autophagy was affected by SIRT1 deacetylation. SIRT1 signaling to DNA damage through PP4 ensures the normal progress of DNA damage repair, which will be beneficial to neuronal regeneration [74,75,76]. SIRT1 plays an important role in AD, especially in the regulation of mitochondrial homeostasis through deacetylation [77]. In several studies, SIRT1 has been shown to be linked to the clearance of Aβ and Tau, and deacetylation of SIRT1 is, one, a transcription factor retinoic acid receptor β to mediate the reduction in neurotoxic Aβ deposition in the brain to improve the repair rate of damaged neurons, and, two, may allow ubiquitin ligases to target tau proteins to facilitate the clearance of these proteins rather than allowing for their pathological intracellular aggregation [78,79,80]. Therefore, deacetylation of SIRT1 has been shown to protect neurons in AD and enhance cognition [81].

Peroxisome proliferator-activated receptor coactivator 1α (PGC-1α) is a major transcriptional coactivator that regulates mitochondrial function and maintains mitochondrial homeostasis. As a semi-autonomous organelle, the downstream target of PGC-1α, TFAM, acts as a communication substance between the nucleus and the mitochondrial nucleus, regulating mitochondrial fusion and division. NAD^+^ can indirectly activate PGC-1α from multiple pathways; firstly, movement can activate AMPK to activate PGC-1α via phosphorylation, and, secondly, it can be activated by altered NAD^+^/NADH SIRT1, which, in turn, deacetylates and activates PGC-1α, which is involved in the regulation of metabolic homeostasis and mitochondrial function, increasing mitochondrial biosynthesis and oxygen consumption [82]. It has been found that PGC-1α is associated with mitochondrial autophagy. When PGC-1α is activated, Nrf1 in the nucleus enters the mitochondria, and Nrf1 in the mitochondria returns to the nucleus to activate TFAM, which comes from the nucleus to the mitochondria to regulate mitochondrial biogenesis [83,84].

When PGC-1α is activated, the mitochondria are more inclined to fuse, i.e., Mfn1/2 is activated. It has been demonstrated that MFN2 deficiency reduces the autophagic activity in energy-stressed cells, suggesting that PGC-1α can mediate mitochondrial autophagy through mitochondrial biogenesis [85]. Mitochondrial autophagy protects the neurons in AD patients, and PGC-1α may reduce Aβ load by regulating BACE1 ubiquitination and degradation. Thus, increased NAD^+^ may play a therapeutic role in AD by reducing BACE1 levels [86]. Katouri et al. further demonstrated the neuroprotective effects of PGC-1α by transferring PGC-1a to the cortical and hippocampal CA1 regions of AD mice using a lentiviral vector, which demonstrated that the upregulation of PGC-1α can improve mitochondrial dynamics, as well as spatial memory and cognitive function, and it can prevent neuronal loss [87].

### 3.3. Exercise Ameliorates Abnormal Mitochondrial Autophagy in AD

AMPK acts as a sensor of the energy metabolism and can receive stimuli from changes in the AMP/ATP ratio. Changes in the levels of ATP, ADP, and AMP activate AMPK [88]. In addition to this, exercise activates AMPK by changing the NAD^+^/NADH ratio [89].

**Figure 2:**
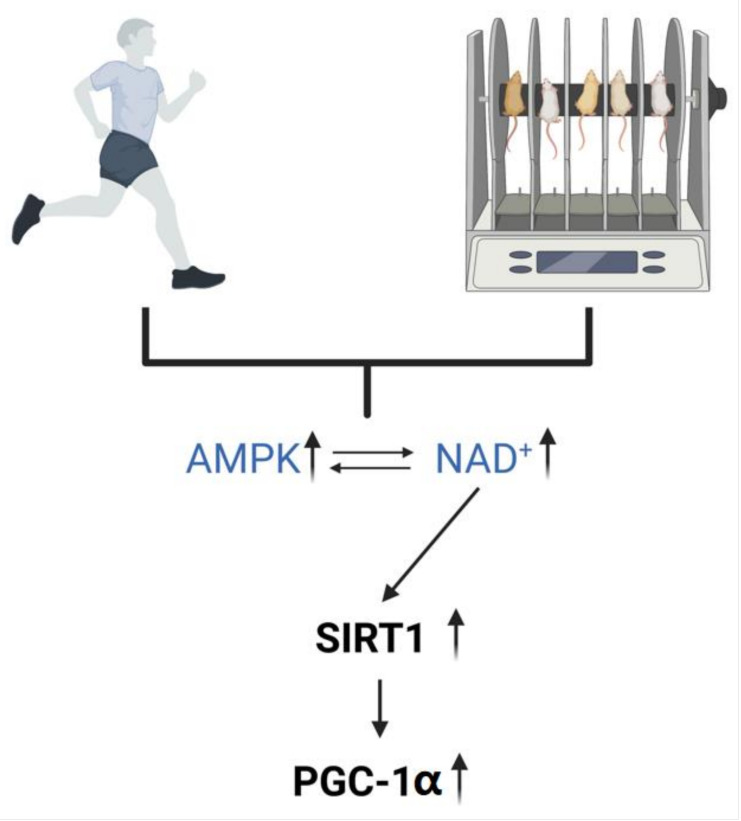
Exercise upregulates AMPK and NAD^+^ to activate SIRT1 and PGC-1α. Exercise in humans and rodents drives the upregulation of NAD^+^ and AMPK and activates the AMPK–SIRT1–PGC-1α signaling pathway.

In our laboratory, we found that aerobic exercise activates AMPK in the brain of APP/PS1 transgenic mice [52]. AMPK has multiple effects on mitochondria, both regulating the rate of mitochondrial ATP production to control the rate of energy metabolism and activating SIRT1 to phosphorylate PGC-1α to mediate mitochondrial dynamics, restore mitochondrial function, and increase mitochondrial activity (Figure 2). It is suggested that improved mitochondrial autophagy provides energy for the clearance of Aβ and tau [90].

Exercise mediates different mitochondrial autophagic pathways through AMPK. First, the AMPK–SIRT1–PGC-1α pathway, in which AMPK and SIRT1 have close interactions in energy regulation, metabolism, and aging, as they can mutually enhance one another’s activities [91,92]. NAD^+^ enhances SIRT1 activity by activating AMPK, leading to deacetylation of PGC-1α, a downstream target of SIRT1, thus activating the AMPK–SIRT1–PGC-1α signaling pathway [93,94]. Therefore, exercise can improve mitochondrial health through mitochondrial biogenesis and the removal of damaged/dysfunctional mitochondria through mitochondrial autophagy [95,96,97].

The second is the AMPK–ULK1 mitochondrial autophagy pathway. Experiments have shown that ULK1 can be activated directly after the upregulation of AMPK by exercise [98]. In this experiment, by using the novel fluorescent reporter gene pMitoTimer, monitoring revealed that mice experienced mitochondrial oxidative stress 3–12 h after acute treadmill exercise and mitochondrial autophagy 6 h after skeletal muscle exercise. Exercise-induced metabolic adaptation requiring ulk1 was proven in the same experiment. These findings provide direct evidence of exercise-induced mitochondrial phagocytosis and demonstrate the importance of AMPK–Ulk1 signaling in skeletal muscle [98].

The third is the AMPK–TBK1 mitochondrial autophagy pathway. Upregulation of AMPK can also directly activate TBK1 to mediate mitochondrial autophagy, thus, independent of the PINK–PARKIN mitochondrial autophagy pathway. In a 2020 experiment, increased phosphorylation of TANK-binding kinase 1 (TBK1) in the absence of PINK1 was demonstrated in a non-muscle cell line, regulated by AMPK-dependent signaling. TBK1 activation by AMPK mediates mitochondrial autophagy by phosphorylating P52, P62, and OPTN, while TBK1 can control mitochondrial mass in a manner that regulates cell growth by isolating centrosomes to affect cell mitosis [99].

The fourth is the AMPK–MFF–TBK1 mitochondrial autophagy pathway. AMPK also promotes mitochondrial autophagy by activating MFF phosphorylation to enhance mitochondrial fission and by activating TBK1 to promote autophagosomal phagocytosis [100]. During PINK1/Parkin-mediated mitochondrial autophagy, TBK1 is directly or indirectly mediated by phosphorylation of the autophagy receptors [30]. TBK1 activity is required for efficient recruitment of OPTN and NDP52 to ubiquitinated mitochondria, where TBK1 phosphorylates OPTN at Ser177 to increase the LC3 binding affinity and at Ser473 and Ser513 to further increase binding of OPTN to the ubiquitin chain [101,102]. Therefore, in addition to the Parkin–PINK1 mitochondrial autophagy pathway, another mitochondrial autophagy pathway (ubiquitin–OPTN–TBK1), constitutes more landing sites for autophagy joints on damaged mitochondria.

## 4. Potential Mechanisms for Upregulation of NAD^+^ to Improve Mitochondrial Autophagy

### 4.1. SLC12A8—An NMN Transporter Protein on the Cell Membrane

SLC12A8 is a solute carrier responsible for material transport across cell membranes [103]. Nearly 100 human SLCs have been proposed to transport amino acids, 60% of which have been shown to transport amino acids, while the rest are closely related to phylogenetically known amino acid transporter proteins [104]. In an earlier study, by examining 195 psoriasis families from Sweden, associations with five marker haplotypes were identified, including haplotype spanning member 8 of the solute carrier family 12 (SLC12A8) [105].

In 2019, in a study by Alessia Grozio et al., experiments were first performed by studying SLC12A8 in mouse liver. To exclude interference with experimental measurements following CD73-mediated degradation of extracellular NMN to NR, followed by re-synthesis of NR into cells by NAMPT, these were excluded using inhibitors (inhibition of NR entry via the nucleoside transporter and inhibition of NAMPT-mediated intracellular NMN synthesis). Then, 100 μM of NMN was added, and the intracellular NMN levels were found to be significantly elevated at the 1 min time point in primary mouse hepatocytes compared to controls. Under these conditions, NMN uptake in primary hepatocytes was examined using the same inhibitor and 100 μM of NMN in stem cells knocked down for SLC12A8 and Nrk1 (a major NR kinase that converts NR to NMN intracellularly) (knockdown efficiency of approximately 80% for both genes) and at the 1 min time point. The rapid uptake of NMN was completely eliminated in SLC12A8 knockout (SLC12A8-KD) hepatocytes, whereas no significant reduction in NMN uptake was observed in Nrk 1 knockout (Nrk 1-KD) hepatocytes, suggesting that SLC12A8 is required for rapid NMN uptake in primary hepatocytes and that the observed increase in intracellular NMN was not due to the conversion of NR or nicotinamide to NMN. Additionally, in experiments, SLC12A8 was found to be expressed in the liver, small intestine, and hippocampal neurospheres, with high expression in the small intestine and pancreas and moderate expression in the liver and white adipose tissue [19].

In a paper titled “SlC12A8 in the lateral hypothalamus maintains energy metabolism and skeletal muscle function in aging” published by Naoki Ito et al. in July 2022, it was found that SCL12A8 expression is also present in the cells in the hypothalamus, and its overexpression effectively regulates hypothalamic function, thereby improving the energy metabolism and skeletal muscle function reduced by aging. It also allows the hypothalamus to regulate glycolysis through protein synthesis to regulate skeletal muscle mass and modulate the sympathetic–β2-adrenergic receptor (β2AR) axis in skeletal muscle [106].

**Figure 3:**
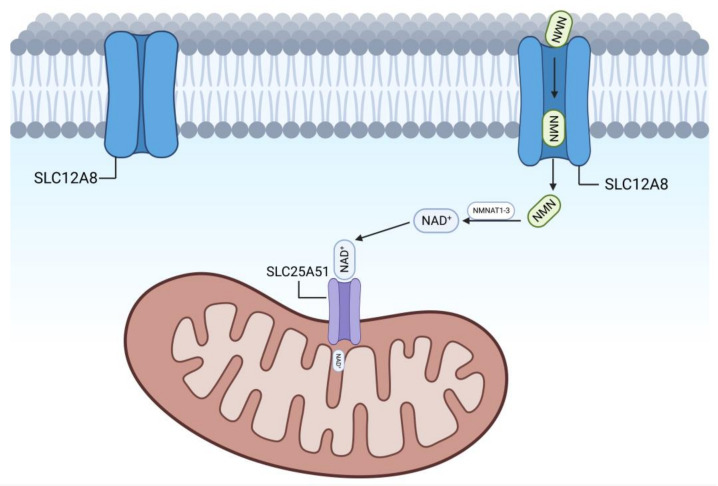
Related transporter proteins on the cell membrane and mitochondrial outer membrane. Extracellular NMN enters the cell interior through SLC12A8 on the cell membrane, is synthesized into NAD^+^ by NAMPT, and then is transported into the mitochondrial lumen through SLC25A51 on the mitochondrial outer membrane to participate in the TCA cycle.

In summary, SLC12A8 is now identified as a transporter protein for NMN. In Figure 3, SLC12A8 is located on the cell membrane (Figure 3).

### 4.2. SLC25A51—An NAD^+^ Transporter Protein on Mitochondria

For many years after 1996, it was thought that there was no NAD^+^ transporter protein in the cell membrane, an idea supported by an in vitro experiment on mitochondria extracted from rat liver cells, which showed that NAD^+^ does not cross the inner membrane of mitochondria [107]. The lack of NAD^+^ transport between cytoplasm and mitochondria is supported by data showing that mitochondrial NAD^+^ is maintained within normal physiological concentrations. After treatment of cells with the DNA alkylating agent MMS or inhibition of NAMPT activity with the inhibitor FK866, NAD^+^ in cytoplasm and nucleus was significantly depleted, but mitochondrial NAD^+^ was maintained within normal physiological concentrations, supporting the absence of NAD^+^ transport between the cytoplasm and mitochondria [108,109]. The only alternative pathway for NAD^+^ import from the cytoplasm appears to be intra-mitochondrial synthesis.

However, in 2020, three independent experimental groups all confirmed the presence of a transporter protein, SLC25A51, on the inner mitochondrial membrane that transports NAD^+^ into the mitochondria. In October 2020, Nora et al. stated that SLC25A51 is required for mitochondrial NAD^+^ transport [20]. SLC25A51 belongs to the same family as SLC12A8, mentioned in the previous section. First, Nora et al. identified a previously unstudied gene, SLC25A51, through sequencing, and found that SLC25A51 is localized to the inner mitochondrial membrane in Hela cells, after immunofluorescence detection and STED microscopy. Subsequently, to test whether SLC25A51 is involved in the mitochondrial energy metabolism, SLC25A51 was knocked out in Hela cells. A defect in mitochondrial function was found in Hela cells by culture, in which SLC25A51 was knocked out and had a significantly lower OCR and reduced total cellular ATP levels, indicating an impaired mitochondrial energy metabolism. Interestingly, SLC25A51 deletion causes defects in the mitochondrial metabolism and ETC complex I activity but does not affect mitochondrial integrity, unlike the usual situation of an impaired mitochondrial respiration rate, which is usually due to defects in mitochondrial replication, translation, or structural integrity, resulting in loss of respiratory chain complexes. However, loss of SLC25A51 does not alter the morphology of mitochondria or mitochondrial ridges, nor does it alter mitochondrial DNA or mass. In addition, the mitochondrial membrane potential and the levels of mitochondrial and nuclear-encoded mitochondrial proteins are only slightly affected, so in the next experiments, it was found that the reduced respiratory rate following SLC25A51 deletion was caused by the loss of intracellular mitochondrial metabolites but probably due to the presence of cytoplasmic lysates that replenished the lost metabolites and, thus, did not cause structural damage to the mitochondria [20].

In December 2020, Enrico et al. similarly concluded that SLC25A51 is an NAD^+^ transporter protein located on the inner mitochondrial membrane [110]. Their team first found a strong correlation with SLC2A1, a glucose transporter protein expressed at the plasma membrane and a major regulator of glycolytic metabolism, by performing a genetic interaction analysis of SLC25A51 with other SLC families located on the inner mitochondrial membrane, suggesting a correlation between SLC25A51 and mitochondrial energy metabolism. SLC25A51, in turn, has coding complementarity with SLC25A3 and is functionally related but not non-redundantly functional. Co-efficient analysis of SCL25A51 and SLC25A3 was then performed to determine their important role in the energy metabolism. Subsequently, by comparing whole-cell and mitochondrial-targeted metabolomics, knockout of SLC25A51 was identified as the key to affect energy metabolites, and it was found that NAD^+^ was the only molecule significantly depleted in SLC25A51 KO cells, and the intracellular NAD^+^ level was restored after SLC25A51 overexpression [110].

To further identify SLC25A51 as an NAD^+^ transporter protein on mammalian mitochondrial membranes, Ndt1 and Ndt2, previously identified on mitochondrial membranes in yeast cells, were used [111]. Enrico et al. implanted yeast Ndt1 into SLC25A51-deficient cells and found that it reversed the mitochondrial respiration defect in these cells [110].

Timothy et al.’s team similarly screened several channel proteins on the mitochondrial membrane, including SLC25A51 and SLC25A52, and concluded that SLC25A51 is the channel protein that is primarily a transporter of NAD^+^ on the mitochondrial membrane. The NAD^+^ content inside the mitochondria of SLC25A51 KO cells was significantly reduced, and mitochondrial respiration was severely affected by the culture of SLC25A51 KO cells compared with normal cells, but interestingly, the whole-cell NAD^+^/NADH was not altered, and the mitochondrial membrane potential was not significantly altered. After re-expression of SLC25A51 in SLC25A51KO cells, the NAD^+^ content in cellular mitochondria was restored [112]. All of the above experiments demonstrated that SLC25A51 is a NAD^+^ transporter protein located on the mitochondrial outer membrane (Figure 3).

### 4.3. SLC12A8 and SLC25A51 May Be Potential Therapeutic Targets for Improving Mitochondrial Autophagy

As described in the previous section, many experiments in recent years have shown that exercise can upregulate NAD^+^, AMPK, SIRT1, and PGC-1α and can effectively improve the mitochondrial membrane potential, mitochondrial ridge, mitochondrial dynamics, and mitochondrial autophagy. Here, we may propose the hypothesis that exercise increases cellular NMN uptake by increasing SLC12A8 in the cell membrane and SLC25A51 in the mitochondrial membrane, the uptake of extracellular NMN to increase intracellular NMN content, and the mitochondrial uptake of intracellular NAD^+^. Intracellular NMN is converted to NAD^+^ by NMNAT1-3, and upregulation of intracellular NAD^+^ activates the AMPK–SIRT1–PGC-1α signaling pathway to improve mitochondrial dynamics (mitochondrial fusion and division), which, in turn, mediates the PINK1/Parkin mitochondrial autophagy signaling pathway via Mnf1, thereby improving mitochondrial autophagy.

**Figure 4:**
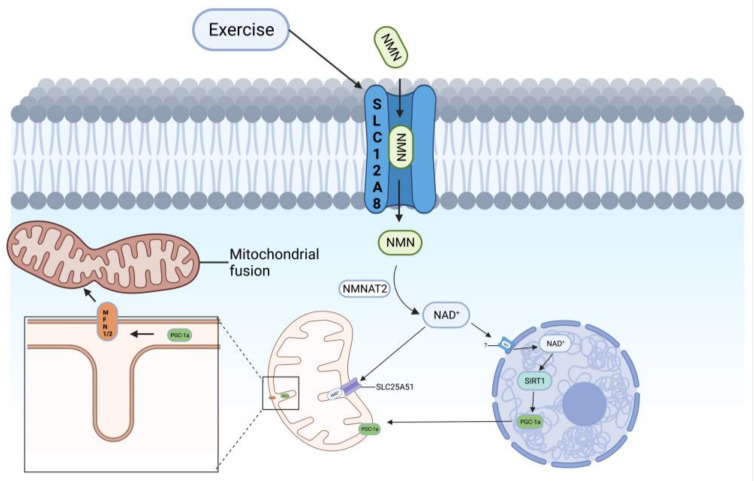
Possible mechanisms by which exercise upregulates NAD^+^ to improve mitochondrial autophagy. Exercise promotes SLC12A8 expression, which allows more NMN to enter the cell membrane from the extracellular fluid, and NMNAT2 in the cytoplasm converts NMN to NAD^+^. NAD^+^ in the cytoplasm can enter the nucleus or the mitochondria via SLC25A51. NAD^+^ entering the mitochondria activates the SIRT1–PGC-1α signaling pathway. Transfer of PGC-1α from the nucleus to the mitochondria activates Mfn1/2 to mediate mitochondrial fusion and improve mitochondrial autophagy.

In the previous section, we mentioned that exercise upregulates NAD^+^, AMPK, and SIRT1 in vivo. Therefore, we propose the following hypothesis: is the expression of SLC12A8, a channel protein of NMN as a NAD^+^ precursor, activated by exercise (Figure 4), so that more NMN will enter the cell and then be converted into NAD^+^ by intracellular NMNAT, thus enhancing the NAD^+^ content in vivo? PINK1/Parkin, a mitochondrial autophagy signaling pathway, or PGC-1α and FoXo improve the toxicity of Aβ and Tau, spatial memory, and cognitive memory and prevent neuronal loss. In the latest study, this year, Ryu et al. used NR and caffeine in humans and on different types of cells and found that NR and caffeine can help the aging body to improve NAD^+^ and bioenergy metabolism temporarily, but there seems to be no substantial improvement in long-term cellular energy metabolism, which may be due to the lack of long-term use. These results suggest that NR and caffeine may alter the metabolism of NAD^+^ and bioenergy in the aging body in nature. It may be necessary to improve the degraded NAD^+^ metabolism in the aging body through long-term stimulation, such as periodic regular exercise, so as to improve the spatial memory, cognitive memory, and prevent the loss of neurons in AD [113].

Whether or not SLC12A8, which acts as a Na^+^-dependent transporter protein, and exercise can regulate the Na^+^ concentrations inside and outside myocytes via the sodium–potassium pump, which would promote the turning on of SLC12A8, is unknown, but in cellular experiments, the removal of Na^+^ resulted in a significant decrease in SLC12A8 expression, leading to a decrease in intracellular NAD^+^ content [19,114]. It is suggested that the effect of exercise on Na^+^ may have a regulatory effect on SLC12A8, allowing more NAD^+^ outside the cell to enter the cell to participate in the energy metabolism, DNA repair, chromatin remodeling, cellular senescence, and immune cell function.

## 5. Summary and Outlook

There have been many studies showing that exercise upregulates NAD^+^ levels in vivo, and there are also many studies showing that oral administration or injection of the NAD^+^ precursors NMN or NR can effectively improve the NAD^+^ levels that are downregulated due to aging, thus extending the life span of the organism.

The deficiency of NAD^+^, a key coenzyme in the tricarboxylic acid cycle, will cause mitochondrial dysfunction and lead to abnormalities in the organism. Improving the NAD^+^ levels in the body to treat mitochondrial dysfunction has been demonstrated, so exercise as an inexpensive prescription for its mediated elevation of NAD^+^ can effectively improve impaired mitochondrial function.

At present, the NMN (NAD^+^ precursor) transporter protein SLC12A8, located on the cell membrane, and the NAD^+^ transporter protein SLC25A51, on the mitochondrial membrane, have been identified, which provides new therapeutic avenues to elevate the NAD^+^ levels in cells and mitochondria. This is coupled with the recent discovery of a strong link between gut microbes and body functions [115]. It has been shown that mycoplasma contributes to host NAD^+^ biosynthesis, and experimentally, mycoplasma plays a role similar to that of resistance to NAMPT inhibitors in cancer cells and xenograft tumors. To further verify whether these results are true, researchers have used stable isotope tracing and microbiota-depleted mice, experimentally demonstrating that this bacteria-mediated deamidation contributed significantly to the NAD^+^-enhancing effects of oral nicotinamide and nicotinamide riboside supplements in several tissues. The findings revealed an important role for the bacterially enabled deamidation pathway in host NAD^+^ metabolism [115]. In our laboratory’s research on the relationship between AD and gut microbiota, it has been found that brain inflammation in APP/PS1 mice is closely related to gut microbial metabolites and bacterial lipopolysaccharide (LPS), and exercise enriches gut microbial diversity and alleviates neuroinflammation in the brain. These results suggest that long-term exercise can effectively regulate gut microbiota and the gut barrier, thereby reducing LPS translocation and ultimately alleviating AD-related neuroinflammation [116].

In summary, for the time being, drugs related to NAD^+^ are agonists of NMN, NR, and NAMPT, while drugs related to the activation or overexpression of NMN and NAD^+^ transport proteins have not been studied. Similarly, research on NAD^+^ production by gut microbes is scarce. Therefore, similar probiotic supplements and NMN/NAD^+^ transporter activators could be developed in the future to increase NAD^+^ production and uptake in vivo, thus providing a new therapeutic approach to rescue mitochondrial autophagy abnormalities due to aging, improve mitochondrial function, restore mitochondrial autophagy, and enhance AD neuronal plasticity.

## Data Availability

Not applicable.

## References

[1] Jack C.R. (2022). Advances in Alzheimer’s disease research over the past two decades. Lancet Neurol..

[2] Swerdlow R.H., Khan S.M. (2004). A “mitochondrial cascade hypothesis” for sporadic Alzheimer’s disease. Med. Hypotheses.

[3] Duyckaerts C., Delatour B., Potier M.C. (2009). Classification and basic pathology of Alzheimer disease. Acta Neuropathol..

[4] Manczak M., Kandimalla R., Yin X., Reddy P.H. (2018). Hippocampal mutant APP and amyloid beta-induced cognitive decline, dendritic spine loss, defective autophagy, mitophagy and mitochondrial abnormalities in a mouse model of Alzheimer’s disease. Hum. Mol. Genet..

[5] Cheignon C., Tomas M., Bonnefont-Rousselot D., Faller P., Hureau C., Collin F. (2018). Oxidative stress and the amyloid beta peptide in Alzheimer’s disease. Redox Biol..

[6] Ghavami S., Shojaei S., Yeganeh B., Ande S.R., Jangamreddy J.R., Mehrpour M., Christoffersson J., Chaabane W., Moghadam A.R., Kashani H.H. (2014). Autophagy and apoptosis dysfunction in neurodegenerative disorders. Prog. Neurobiol..

[7] Bieganowski P., Brenner C. (2004). Discoveries of nicotinamide riboside as a nutrient and conserved NRK genes establish a Preiss-Handler independent route to NAD^+^ in fungi and humans. Cell.

[8] Ramanathan C., Lackie T., Williams D.H., Simone P.S., Zhang Y., Bloomer R.J. (2022). Oral Administration of Nicotinamide Mononucleotide Increases Nicotinamide Adenine Dinucleotide Level in an Animal Brain. Nutrients.

[9] Chini C.C.S., Tarragó M.G., Chini E.N. (2017). NAD and the aging process: Role in life, death and everything in between. Mol. Cell. Endocrinol..

[10] Migliavacca E., Tay S.K.H., Patel H.P., Sonntag T., Civiletto G., McFarlane C., Forrester T., Barton S.J., Leow M.K., Antoun E. (2019). Mitochondrial oxidative capacity and NAD(+) biosynthesis are reduced in human sarcopenia across ethnicities. Nat. Commun..

[11] Covarrubias A.J., Perrone R., Grozio A., Verdin E. (2021). NAD(+) metabolism and its roles in cellular processes during ageing. Nat. Rev. Mol. Cell Biol..

[12] Klimova N., Kristian T. (2019). Multi-targeted Effect of Nicotinamide Mononucleotide on Brain Bioenergetic Metabolism. Neurochem. Res..

[13] Klimova N., Fearnow A., Long A., Kristian T. (2020). NAD(+) precursor modulates post-ischemic mitochondrial fragmentation and reactive oxygen species generation via SIRT3 dependent mechanisms. Exp. Neurol..

[14] Fang E.F., Hou Y., Palikaras K., Adriaanse B.A., Kerr J.S., Yang B., Lautrup S., Hasan-Olive M.M., Caponio D., Dan X. (2019). Mitophagy inhibits amyloid-β and tau pathology and reverses cognitive deficits in models of Alzheimer’s disease. Nat. Neurosci..

[15] Fang E.F. (2019). Mitophagy and NAD(+) inhibit Alzheimer disease. Autophagy.

[16] De la Rosa A., Olaso-Gonzalez G., Arc-Chagnaud C., Millan F., Salvador-Pascual A., García-Lucerga C., Blasco-Lafarga C., Garcia-Dominguez E., Carretero A., Correas A.G. (2020). Physical exercise in the prevention and treatment of Alzheimer’s disease. J. Sport Health Sci..

[17] Chong M.C., Silva A., James P.F., Wu S.S.X., Howitt J. (2022). Exercise increases the release of NAMPT in extracellular vesicles and alters NAD(+) activity in recipient cells. Aging Cell.

[18] Ryu K.W., Nandu T., Kim J., Challa S., DeBerardinis R.J., Kraus W.L. (2018). Metabolic regulation of transcription through compartmentalized NAD(+) biosynthesis. Science.

[19] Grozio A., Mills K.F., Yoshino J., Bruzzone S., Sociali G., Tokizane K., Lei H.C., Cunningham R., Sasaki Y., Migaud M.E. (2019). Slc12a8 is a nicotinamide mononucleotide transporter. Nat Metab..

[20] Kory N., Uit de Bos J., van der Rijt S., Jankovic N., Güra M., Arp N., Pena I.A., Prakash G., Chan S.H., Kunchok T. (2020). MCART1/SLC25A51 is required for mitochondrial NAD transport. Sci. Adv..

[21] Wang T., Zhang F., Peng W., Wang L., Zhang J., Dong W., Tian X., Ye C., Li Y., Gong Y. (2022). Overexpression of NMNAT3 improves mitochondrial function and enhances antioxidative stress capacity of bone marrow mesenchymal stem cells via the NAD+-Sirt3 pathway. Biosci. Rep..

[22] Irie J., Inagaki E., Fujita M., Nakaya H., Mitsuishi M., Yamaguchi S., Yamashita K., Shigaki S., Ono T., Yukioka H. (2020). Effect of oral administration of nicotinamide mononucleotide on clinical parameters and nicotinamide metabolite levels in healthy Japanese men. Endocr. J..

[23] Long A.N., Owens K., Schlappal A.E., Kristian T., Fishman P.S., Schuh R.A. (2015). Effect of nicotinamide mononucleotide on brain mitochondrial respiratory deficits in an Alzheimer’s disease-relevant murine model. BMC Neurol..

[24] Mills K.F., Yoshida S., Stein L.R., Grozio A., Kubota S., Sasaki Y., Redpath P., Migaud M.E., Apte R.S., Uchida K. (2016). Long-Term Administration of Nicotinamide Mononucleotide Mitigates Age-Associated Physiological Decline in Mice. Cell Metab..

[25] Yoshino J., Mills K.F., Yoon M.J., Imai S. (2011). Nicotinamide mononucleotide, a key NAD(+) intermediate, treats the pathophysiology of diet- and age-induced diabetes in mice. Cell Metab..

[26] Peek C.B., Affinati A.H., Ramsey K.M., Kuo H.Y., Yu W., Sena L.A., Ilkayeva O., Marcheva B., Kobayashi Y., Omura C. (2013). Circadian clock NAD^+^ cycle drives mitochondrial oxidative metabolism in mice. Science.

[27] Stromsdorfer K.L., Yamaguchi S., Yoon M.J., Moseley A.C., Franczyk M.P., Kelly S.C., Qi N., Imai S., Yoshino J. (2016). NAMPT-Mediated NAD(+) Biosynthesis in Adipocytes Regulates Adipose Tissue Function and Multi-organ Insulin Sensitivity in Mice. Cell Rep..

[28] Karamanlidis G., Lee C.F., Garcia-Menendez L., Kolwicz S.C., Suthammarak W., Gong G., Sedensky M.M., Morgan P.G., Wang W., Tian R. (2013). Mitochondrial complex I deficiency increases protein acetylation and accelerates heart failure. Cell Metab..

[29] Martin A.S., Abraham D.M., Hershberger K.A., Bhatt D.P., Mao L., Cui H., Liu J., Liu X., Muehlbauer M.J., Grimsrud P.A. (2017). Nicotinamide mononucleotide requires SIRT3 to improve cardiac function and bioenergetics in a Friedreich’s ataxia cardiomyopathy model. JCI Insight.

[30] North B.J., Rosenberg M.A., Jeganathan K.B., Hafner A.V., Michan S., Dai J., Baker D.J., Cen Y., Wu L.E., Sauve A.A. (2014). SIRT2 induces the checkpoint kinase BubR1 to increase lifespan. EMBO J..

[31] Yamamoto T., Byun J., Zhai P., Ikeda Y., Oka S., Sadoshima J. (2014). Nicotinamide mononucleotide, an intermediate of NAD^+^ synthesis, protects the heart from ischemia and reperfusion. PLoS ONE.

[32] Pirinen E., Auranen M., Khan N.A., Brilhante V., Urho N., Pessia A., Hakkarainen A., Kuula J., Heinonen U., Schmidt M.S. (2020). Niacin Cures Systemic NAD(+) Deficiency and Improves Muscle Performance in Adult-Onset Mitochondrial Myopathy. Cell Metab..

[33] Guan Y., Wang S.R., Huang X.Z., Xie Q.H., Xu Y.Y., Shang D., Hao C.M. (2017). Nicotinamide Mononucleotide, an NAD(+) Precursor, Rescues Age-Associated Susceptibility to AKI in a Sirtuin 1-Dependent Manner. J. Am. Soc. Nephrol. JASN.

[34] Stein L.R., Imai S. (2014). Specific ablation of Nampt in adult neural stem cells recapitulates their functional defects during aging. EMBO J..

[35] Yoon M.J., Yoshida M., Johnson S., Takikawa A., Usui I., Tobe K., Nakagawa T., Yoshino J., Imai S. (2015). SIRT1-Mediated eNAMPT Secretion from Adipose Tissue Regulates Hypothalamic NAD^+^ and Function in Mice. Cell Metab..

[36] Liu L., Su X., Quinn W.J., Hui S., Krukenberg K., Frederick D.W., Redpath P., Zhan L., Chellappa K., White E. (2018). Quantitative Analysis of NAD Synthesis-Breakdown Fluxes. Cell Metab..

[37] Petley A., Macklin B., Renwick A.G., Wilkin T.J. (1995). The pharmacokinetics of nicotinamide in humans and rodents. Diabetes.

[38] Igarashi M., Nakagawa-Nagahama Y., Miura M., Kashiwabara K., Yaku K., Sawada M., Sekine R., Fukamizu Y., Sato T., Sakurai T. (2022). Chronic nicotinamide mononucleotide supplementation elevates blood nicotinamide adenine dinucleotide levels and alters muscle function in healthy older men. npj Aging.

[39] Campbell J.M. (2022). Supplementation with NAD(+) and Its Precursors to Prevent Cognitive Decline across Disease Contexts. Nutrients.

[40] Yoo K.H., Tang J.J., Rashid M.A., Cho C.H., Corujo-Ramirez A., Choi J., Bae M.G., Brogren D., Hawse J.R., Hou X. (2021). Nicotinamide Mononucleotide Prevents Cisplatin-Induced Cognitive Impairments. Cancer Res..

[41] Zapata-Pérez R., Tammaro A., Schomakers B.V., Scantlebery A.M.L., Denis S., Elfrink H.L., Giroud-Gerbetant J., Cantó C., López-Leonardo C., McIntyre R.L. (2021). Reduced nicotinamide mononucleotide is a new and potent NAD(+) precursor in mammalian cells and mice. FASEB J. Off. Publ. Fed. Am. Soc. Exp. Biol..

[42] White A.T., Schenk S. (2012). NAD(+)/NADH and skeletal muscle mitochondrial adaptations to exercise. Am. J. Physiology. Endocrinol. Metab..

[43] Graham T., Sjøgaard G., Löllgen H., Saltin B. (1978). NAD in muscle of man at rest and during exercise. Pflug. Arch. Eur. J. Physiol..

[44] Katz A., Sahlin K. (1987). Effect of decreased oxygen availability on NADH and lactate contents in human skeletal muscle during exercise. Acta Physiol. Scand..

[45] Hu Q., Wu D., Walker M., Wang P., Tian R., Wang W. (2021). Genetically encoded biosensors for evaluating NAD(+)/NADH ratio in cytosolic and mitochondrial compartments. Cell Rep. Methods.

[46] Wang Y., Stancliffe E., Fowle-Grider R., Wang R., Wang C., Schwaiger-Haber M., Shriver L.P., Patti G.J. (2022). Saturation of the mitochondrial NADH shuttles drives aerobic glycolysis in proliferating cells. Mol. Cell.

[47] Mao P., Reddy P.H. (2011). Aging and amyloid beta-induced oxidative DNA damage and mitochondrial dysfunction in Alzheimer’s disease: Implications for early intervention and therapeutics. Biochim. Biophys. Acta.

[48] Cunnane S.C., Trushina E., Morland C., Prigione A., Casadesus G., Andrews Z.B., Beal M.F., Bergersen L.H., Brinton R.D., de la Monte S. (2020). Brain energy rescue: An emerging therapeutic concept for neurodegenerative disorders of ageing. Nat. Rev. Drug Discov..

[49] De la Cueva M., Antequera D., Ordoñez-Gutierrez L., Wandosell F., Camins A., Carro E., Bartolome F. (2022). Amyloid-β impairs mitochondrial dynamics and autophagy in Alzheimer’s disease experimental models. Sci. Rep..

[50] Zhu X., Smith M.A., Perry G., Aliev G. (2004). Mitochondrial failures in Alzheimer’s disease. Am. J. Alzheimer’s Dis. Other Dement..

[51] Carbonell F., Zijdenbos A.P., Bedell B.J. (2020). Spatially Distributed Amyloid-β Reduces Glucose Metabolism in Mild Cognitive Impairment. J. Alzheimer’s Dis. JAD.

[52] Jian Y., Yuan S., Yang J., Lei Y., Li X., Liu W. (2022). Aerobic Exercise Alleviates Abnormal Autophagy in Brain Cells of APP/PS1 Mice by Upregulating AdipoR1 Levels. Int. J. Mol. Sci..

[53] Shen H., Hyrc K.L., Goldberg M.P. (2013). Maintaining energy homeostasis is an essential component of Wld(S)-mediated axon protection. Neurobiol. Dis..

[54] Reddy P.H., Yin X., Manczak M., Kumar S., Pradeepkiran J.A., Vijayan M., Reddy A.P. (2018). Mutant APP and amyloid beta-induced defective autophagy, mitophagy, mitochondrial structural and functional changes and synaptic damage in hippocampal neurons from Alzheimer’s disease. Hum. Mol. Genet..

[55] Palikaras K., Lionaki E., Tavernarakis N. (2015). Coordination of mitophagy and mitochondrial biogenesis during ageing in *C. elegans*. Nature.

[56] Fang E.F., Scheibye-Knudsen M., Brace L.E., Kassahun H., SenGupta T., Nilsen H., Mitchell J.R., Croteau D.L., Bohr V.A. (2014). Defective mitophagy in XPA via PARP-1 hyperactivation and NAD(+)/SIRT1 reduction. Cell.

[57] Motta M.C., Divecha N., Lemieux M., Kamel C., Chen D., Gu W., Bultsma Y., McBurney M., Guarente L. (2004). Mammalian SIRT1 represses forkhead transcription factors. Cell.

[58] Eijkelenboom A., Burgering B.M. (2013). FOXOs: Signalling integrators for homeostasis maintenance. Nat. Rev. Mol. Cell Biol..

[59] Miller K.N., Clark J.P., Martin S.A., Howell P.R., Burhans M.S., Haws S.A., Johnson N.B., Rhoads T.W., Pavelec D.M., Eliceiri K.W. (2019). PGC-1a integrates a metabolism and growth network linked to caloric restriction. Aging Cell.

[60] Xiao W., Wang R.S., Handy D.E., Loscalzo J. (2018). NAD(H) and NADP(H) Redox Couples and Cellular Energy Metabolism. Antioxid. Redox Signal..

[61] Katsyuba E., Auwerx J. (2017). Modulating NAD(+) metabolism, from bench to bedside. EMBO J..

[62] Mouchiroud L., Houtkooper R.H., Moullan N., Katsyuba E., Ryu D., Cantó C., Mottis A., Jo Y.S., Viswanathan M., Schoonjans K. (2013). The NAD(+)/Sirtuin Pathway Modulates Longevity through Activation of Mitochondrial UPR and FOXO Signaling. Cell.

[63] Zhang H., Ryu D., Wu Y., Gariani K., Wang X., Luan P., D’Amico D., Ropelle E.R., Lutolf M.P., Aebersold R. (2016). NAD^+^ repletion improves mitochondrial and stem cell function and enhances life span in mice. Science.

[64] Zhu X.H., Lu M., Lee B.Y., Ugurbil K., Chen W. (2015). In vivo NAD assay reveals the intracellular NAD contents and redox state in healthy human brain and their age dependences. Proc. Natl. Acad. Sci. USA.

[65] Yoshino J., Baur J.A., Imai S.I. (2018). NAD(+) Intermediates: The Biology and Therapeutic Potential of NMN and NR. Cell Metab..

[66] Imai S., Yoshino J. (2013). The importance of NAMPT/NAD/SIRT1 in the systemic regulation of metabolism and ageing. Diabetes Obes. Metab..

[67] Yao Z., Yang W., Gao Z., Jia P. (2017). Nicotinamide mononucleotide inhibits JNK activation to reverse Alzheimer disease. Neurosci. Lett..

[68] Michishita E., Park J.Y., Burneskis J.M., Barrett J.C., Horikawa I. (2005). Evolutionarily conserved and nonconserved cellular localizations and functions of human SIRT proteins. Mol. Biol. Cell.

[69] Funk J.A., Odejinmi S., Schnellmann R.G. (2010). SRT1720 induces mitochondrial biogenesis and rescues mitochondrial function after oxidant injury in renal proximal tubule cells. J. Pharmacol. Exp. Ther..

[70] Lee I.H., Cao L., Mostoslavsky R., Lombard D.B., Liu J., Bruns N.E., Tsokos M., Alt F.W., Finkel T. (2008). A role for the NAD-dependent deacetylase Sirt1 in the regulation of autophagy. Proc. Natl. Acad. Sci. USA.

[71] Ou X., Lee M.R., Huang X., Messina-Graham S., Broxmeyer H.E. (2014). SIRT1 positively regulates autophagy and mitochondria function in embryonic stem cells under oxidative stress. Stem Cells Dayt. Ohio.

[72] Liu X., Cai S., Zhang C., Liu Z., Luo J., Xing B., Du X. (2018). Deacetylation of NAT10 by Sirt1 promotes the transition from rRNA biogenesis to autophagy upon energy stress. Nucleic Acids Res..

[73] Lazarou M., Sliter D.A., Kane L.A., Sarraf S.A., Wang C., Burman J.L., Sideris D.P., Fogel A.I., Youle R.J. (2015). The ubiquitin kinase PINK1 recruits autophagy receptors to induce mitophagy. Nature.

[74] Rasti G., Becker M., Vazquez B.N., Espinosa-Alcantud M., Fernández-Duran I., Gámez-García A., Ianni A., Gonzalez J., Bosch-Presegué L., Marazuela-Duque A. (2023). SIRT1 regulates DNA damage signaling through the PP4 phosphatase complex. Nucleic Acids Res..

[75] Xu Y., Wan W. (2023). Acetylation in the regulation of autophagy. Autophagy.

[76] Li X., Cao G., Liu X., Tang T.S., Guo C., Liu H. (2022). Polymerases and DNA Repair in Neurons: Implications in Neuronal Survival and Neurodegenerative Diseases. Front. Cell. Neurosci..

[77] Xu H., Liu Y.Y., Li L.S., Liu Y.S. (2023). Sirtuins at the Crossroads between Mitochondrial Quality Control and Neurodegenerative Diseases: Structure, Regulation, Modifications, and Modulators. Aging Dis..

[78] Shah S.A., Yoon G.H., Chung S.S., Abid M.N., Kim T.H., Lee H.Y., Kim M.O. (2017). Novel osmotin inhibits SREBP2 via the AdipoR1/AMPK/SIRT1 pathway to improve Alzheimer’s disease neuropathological deficits. Mol. Psychiatry.

[79] Min S.W., Cho S.H., Zhou Y., Schroeder S., Haroutunian V., Seeley W.W., Huang E.J., Shen Y., Masliah E., Mukherjee C. (2010). Acetylation of tau inhibits its degradation and contributes to tauopathy. Neuron.

[80] Shin M.K., Vázquez-Rosa E., Koh Y., Dhar M., Chaubey K., Cintrón-Pérez C.J., Barker S., Miller E., Franke K., Noterman M.F. (2021). Reducing acetylated tau is neuroprotective in brain injury. Cell.

[81] Corpas R., Revilla S., Ursulet S., Castro-Freire M., Kaliman P., Petegnief V., Giménez-Llort L., Sarkis C., Pallàs M., Sanfeliu C. (2017). SIRT1 Overexpression in Mouse Hippocampus Induces Cognitive Enhancement Through Proteostatic and Neurotrophic Mechanisms. Mol. Neurobiol..

[82] Kitada M., Ogura Y., Monno I., Koya D. (2019). Sirtuins and Type 2 Diabetes: Role in Inflammation, Oxidative Stress, and Mitochondrial Function. Front. Endocrinol..

[83] Scarpulla R.C. (2008). Transcriptional paradigms in mammalian mitochondrial biogenesis and function. Physiol. Rev..

[84] Gleyzer N., Vercauteren K., Scarpulla R.C. (2005). Control of mitochondrial transcription specificity factors (TFB1M and TFB2M) by nuclear respiratory factors (NRF-1 and NRF-2) and PGC-1 family coactivators. Mol. Cell. Biol..

[85] Hu Y., Chen H., Zhang L., Lin X., Li X., Zhuang H., Fan H., Meng T., He Z., Huang H. (2021). The AMPK-MFN2 axis regulates MAM dynamics and autophagy induced by energy stresses. Autophagy.

[86] Hampel H., Vassar R., De Strooper B., Hardy J., Willem M., Singh N., Zhou J., Yan R., Vanmechelen E., De Vos A. (2021). The β-Secretase BACE1 in Alzheimer’s Disease. Biol. Psychiatry.

[87] Wrann C.D., White J.P., Salogiannnis J., Laznik-Bogoslavski D., Wu J., Ma D., Lin J.D., Greenberg M.E., Spiegelman B.M. (2013). Exercise induces hippocampal BDNF through a PGC-1α/FNDC5 pathway. Cell Metab..

[88] Herzig S., Shaw R.J. (2018). AMPK: Guardian of metabolism and mitochondrial homeostasis. Nat. Reviews. Mol. Cell Biol..

[89] Wiley C.D., Velarde M.C., Lecot P., Liu S., Sarnoski E.A., Freund A., Shirakawa K., Lim H.W., Davis S.S., Ramanathan A. (2016). Mitochondrial Dysfunction Induces Senescence with a Distinct Secretory Phenotype. Cell Metab..

[90] Godoy J.A., Zolezzi J.M., Braidy N., Inestrosa N.C. (2014). Role of Sirt1 during the ageing process: Relevance to protection of synapses in the brain. Mol. Neurobiol..

[91] Sung B., Chung J.W., Bae H.R., Choi J.S., Kim C.M., Kim N.D. (2015). Humulus japonicus extract exhibits antioxidative and anti-aging effects via modulation of the AMPK-SIRT1 pathway. Exp. Ther. Med..

[92] Salminen A., Kaarniranta K., Kauppinen A. (2013). Crosstalk between Oxidative Stress and SIRT1: Impact on the Aging Process. Int. J. Mol. Sci..

[93] Lagouge M., Argmann C., Gerhart-Hines Z., Meziane H., Lerin C., Daussin F., Messadeq N., Milne J., Lambert P., Elliott P. (2006). Resveratrol improves mitochondrial function and protects against metabolic disease by activating SIRT1 and PGC-1alpha. Cell.

[94] Gerhart-Hines Z., Rodgers J.T., Bare O., Lerin C., Kim S.H., Mostoslavsky R., Alt F.W., Wu Z., Puigserver P. (2007). Metabolic control of muscle mitochondrial function and fatty acid oxidation through SIRT1/PGC-1alpha. EMBO J..

[95] Halling J.F., Pilegaard H. (2020). PGC-1α-mediated regulation of mitochondrial function and physiological implications. Appl. Physiol. Nutr. Metab. Physiol. Appl. Nutr. Metab..

[96] Romanello V., Sandri M. (2015). Mitochondrial Quality Control and Muscle Mass Maintenance. Front. Physiol..

[97] Yan Z., Okutsu M., Akhtar Y.N., Lira V.A. (2011). Regulation of exercise-induced fiber type transformation, mitochondrial biogenesis, and angiogenesis in skeletal muscle. J. Appl. Physiol..

[98] Laker R.C., Drake J.C., Wilson R.J., Lira V.A., Lewellen B.M., Ryall K.A., Fisher C.C., Zhang M., Saucerman J.J., Goodyear L.J. (2017). Ampk phosphorylation of Ulk1 is required for targeting of mitochondria to lysosomes in exercise-induced mitophagy. Nat. Commun..

[99] Sarraf S.A., Sideris D.P., Giagtzoglou N., Ni L., Kankel M.W., Sen A., Bochicchio L.E., Huang C.H., Nussenzweig S.C., Worley S.H. (2019). PINK1/Parkin Influences Cell Cycle by Sequestering TBK1 at Damaged Mitochondria, Inhibiting Mitosis. Cell Rep..

[100] Seabright A.P., Fine N.H.F., Barlow J.P., Lord S.O., Musa I., Gray A., Bryant J.A., Banzhaf M., Lavery G.G., Hardie D.G. (2020). AMPK activation induces mitophagy and promotes mitochondrial fission while activating TBK1 in a PINK1-Parkin independent manner. FASEB J. Off. Publ. Fed. Am. Soc. Exp. Biol..

[101] Richter B., Sliter D.A., Herhaus L., Stolz A., Wang C., Beli P., Zaffagnini G., Wild P., Martens S., Wagner S.A. (2016). Phosphorylation of OPTN by TBK1 enhances its binding to Ub chains and promotes selective autophagy of damaged mitochondria. Proc. Natl. Acad. Sci. USA.

[102] Heo J.M., Ordureau A., Paulo J.A., Rinehart J., Harper J.W. (2015). The PINK1-PARKIN Mitochondrial Ubiquitylation Pathway Drives a Program of OPTN/NDP52 Recruitment and TBK1 Activation to Promote Mitophagy. Mol. Cell.

[103] Gagnon K.B., Delpire E. (2013). Physiology of SLC12 transporters: Lessons from inherited human genetic mutations and genetically engineered mouse knockouts. Am. J. Physiology. Cell Physiol..

[104] Lin L., Yee S.W., Kim R.B., Giacomini K.M. (2015). SLC transporters as therapeutic targets: Emerging opportunities. Nat. Rev. Drug Discov..

[105] Bowcock A.M., Cookson W.O. (2004). The genetics of psoriasis, psoriatic arthritis and atopic dermatitis. Hum. Mol. Genet..

[106] Ito N., Takatsu A., Ito H., Koike Y., Yoshioka K., Kamei Y., Imai S.I. (2022). Slc12a8 in the lateral hypothalamus maintains energy metabolism and skeletal muscle functions during aging. Cell Rep..

[107] Barile M., Passarella S., Danese G., Quagliariello E. (1996). Rat liver mitochondria can synthesize nicotinamide adenine dinucleotide from nicotinamide mononucleotide and ATP via a putative matrix nicotinamide mononucleotide adenylyltransferase. Biochem. Mol. Biol. Int..

[108] Yang H., Yang T., Baur J.A., Perez E., Matsui T., Carmona J.J., Lamming D.W., Souza-Pinto N.C., Bohr V.A., Rosenzweig A. (2007). Nutrient-sensitive mitochondrial NAD^+^ levels dictate cell survival. Cell.

[109] Pittelli M., Formentini L., Faraco G., Lapucci A., Rapizzi E., Cialdai F., Romano G., Moneti G., Moroni F., Chiarugi A. (2010). Inhibition of nicotinamide phosphoribosyltransferase: Cellular bioenergetics reveals a mitochondrial insensitive NAD pool. J. Biol. Chem..

[110] Girardi E., Agrimi G., Goldmann U., Fiume G., Lindinger S., Sedlyarov V., Srndic I., Gürtl B., Agerer B., Kartnig F. (2020). Epistasis-driven identification of SLC25A51 as a regulator of human mitochondrial NAD import. Nat. Commun..

[111] Todisco S., Agrimi G., Castegna A., Palmieri F. (2006). Identification of the mitochondrial NAD^+^ transporter in Saccharomyces cerevisiae. J. Biol. Chem..

[112] Luongo T.S., Eller J.M., Lu M.J., Niere M., Raith F., Perry C., Bornstein M.R., Oliphint P., Wang L., McReynolds M.R. (2020). SLC25A51 is a mammalian mitochondrial NAD(+) transporter. Nature.

[113] Ryu W.I., Shen M., Lee Y., Healy R.A., Bormann M.K., Cohen B.M., Sonntag K.C. (2022). Nicotinamide riboside and caffeine partially restore diminished NAD availability but not altered energy metabolism in Alzheimer’s disease. Aging Cell.

[114] Wyckelsma V.L., McKenna M.J. (2016). Effects of Age on Na(+),K(+)-ATPase Expression in Human and Rodent Skeletal Muscle. Front. Physiol..

[115] Shats I., Williams J.G., Liu J., Makarov M.V., Wu X., Lih F.B., Deterding L.J., Lim C., Xu X., Randall T.A. (2020). Bacteria Boost Mammalian Host NAD Metabolism by Engaging the Deamidated Biosynthesis Pathway. Cell Metab..

[116] Yuan S., Yang J., Jian Y., Lei Y., Yao S., Hu Z., Liu X., Tang C., Liu W. (2022). Treadmill Exercise Modulates Intestinal Microbes and Suppresses LPS Displacement to Alleviate Neuroinflammation in the Brains of APP/PS1 Mice. Nutrients.

